# Population-Level Preparedness About Preventive Practices Against Coronavirus Disease 2019: A Cross-Sectional Study Among Adults in Bangladesh

**DOI:** 10.3389/fpubh.2020.582701

**Published:** 2021-01-11

**Authors:** Mohammad Bellal Hossain, Md. Zakiul Alam, Md. Syful Islam, Shafayat Sultan, Md. Mahir Faysal, Sharmin Rima, Md. Anwer Hossain, Maliha Mubashirah Mahmood, Shaima Shohuda Kashfi, Abdullah Al Mamun, Hossna Tasmia Monia, Sharmin Sultana Shoma

**Affiliations:** ^1^Department of Population Sciences, University of Dhaka, Dhaka, Bangladesh; ^2^Department of Population Science, Jatiya Kabi Kazi Nazrul Islam University, Mymensingh, Bangladesh; ^3^Ovibashi Karmi Unnayan Program (OKUP), Dhaka, Bangladesh

**Keywords:** Bangladesh, practices, prevention, preparedness, COVID-19, population-level

## Abstract

This study assessed the preparedness regarding the preventive practices toward the coronavirus disease 2019 (COVID-19) among the adult population in Bangladesh. Data were collected through an online survey with a sample size of 1,056. We constructed four variables (individual, household, economic, and community and social distancing) related to preparedness based on the principal component analysis of eight items. We employed descriptive statistics and multiple linear regression analysis. The results showed that the accuracy rate of the overall preparedness scale was 68.9%. The preparedness level related to economic, individual, household, and community and social distancing was 64.9, 77.1, 50.4, and 83.2%, respectively. However, the economic preparedness significantly varied by sex, education, occupation, attitude, and worries related to COVID-19. Individual preparedness was significantly associated with education, residence, and attitudes. The household preparedness significantly varied by education, residence, and worries, while the respondent's community and social distancing-related preparedness significantly varied by sex, region, residence, and attitude. This study implies the necessity of the coverage of financial schemes for the vulnerable group. Increased coverage of health education regarding personal hygiene targeting the less educated and rural population should be ensured.

## Introduction

The pandemic of novel coronavirus disease 2019 (COVID-19) is spreading rapidly across Bangladesh. The first case of COVID-19 in Bangladesh was confirmed on 8 March 2020. Bangladesh was having a slow and steady increase in the overall COVID-19 attack rate (AR) in the first 2 months. However, the transmission of the virus is increasing very rapidly since the beginning of the third month. The average AR was only 1.0 per million population for the first month (7 April), which increased to 73.6 in the second month (7 May), followed by 389.5 (7 June) and 998.8 (7 July) in the third and fourth months, respectively (1). The total number of COVID-19 positive cases was 4,28,965 as of 13 November 2020 (1). Against these confirmed cases, reported death rates have reached 1.3% of the infected persons.

The increased numbers of COVID-19 positive persons and death rates have created massive pressure on the already fragile health systems of Bangladesh. Thus, the country has adopted several non-therapeutic measures in the absence of vaccine and treatment to flatten the curve of the infection and death rates, which included (a) declaring mass lockdown and public holiday (started from 26 March and ended on 31 June); (b) risk zone-based lockdowns (started from 9 June); (c) limited working hours (started from 31 May); and (d) maintaining social isolation protocol and restricting population movement through travel bans (started from 26 March and ended on 31 May) (2, 3). The primary aim of these non-therapeutic measures was to adopt preventive measures against the COVID-19. However, these state-level initiatives in Bangladesh were not effective enough to ensure preventive practices among the mass population because of their socioeconomic structure and controversies surrounding some specific policy decisions (4, 5). The Chinese experience shows that the adoption of strict preventive practices against COVID-19, such as avoiding crowded places and the mandatory wearing of masks, is dependent on the risk perception, knowledge regarding COVID-19, and the implementation of stringent prevention and control measures by the local governments (6). The studies conducted elsewhere on non-COVID-19-related diseases, and natural disasters show that the socioeconomic situation of the mass population also determined the individual level preparedness, which ultimately influenced them to adopt preventive practices (7–11).

Pandemic preparedness, be it related to the health system, individual, or household level, is one of the critical concerns across the countries for reducing the risk of COVID-19 (12). Thus, research on preparedness and preventive practices related to COVID-19 have significant public health policy implications, as preparedness is the key to navigating any public health crisis (13). A study conducted in Bangladesh shows that the country severely lacked the pandemic preparedness in its health and governance system. This study reported that lack of preparedness due to the “absence of planning and coordination, disproportionate resource allocations, challenged infrastructure, adherence to bureaucratic delay, lack of synchronized risk communication, failing leadership of concerned authorities, and incoherent decision-making” (14) had increased the country's epidemiologic vulnerability. However, no study was conducted to assess preparedness against the COVID-19 in Bangladesh at the individual and household levels, though research conducted elsewhere found that preparedness plays a significant role in adopting preventive practices (15). On the other hand, in Bangladesh, few studies have been conducted to explore the practices toward COVID-19. The findings of these studies show that different precarious practices such as not adopting protective measures and hygiene protocols, not wearing face masks in public places, and not maintaining social distance are prominent among mass population in Bangladesh (3, 16). Thus, the current study aimed to assess the preparedness regarding the preventive practices toward the COVID-19 among the adult population in Bangladesh using an online survey.

## Materials and Methods

### Data Source

We conducted the survey using a cross-sectional research design. Population aged 18 years and above, living in Bangladesh, and who can read and write and use the internet were the criteria for selecting respondents. In Bangladesh, 74.7% of people aged 15 years and above can read and write a short, simple statement about their everyday life (17). On the other hand, as of March 2020, about 61% of the population are internet users in Bangladesh (18). We developed the study questionnaire based on the guidelines for conducting the behavioral insights on COVID-19 by the Regional Office for Europe of the World Health Organization (WHO) (19). The tool was adapted and customized for the Bangladesh country context. The tool was then translated into Bengali (local language) and pretested. The WHO (19) recommended having a sample size of 1,000 adult population. The data for this study were collected from 10 to 16 May 2020. The country was partially locked down during this period, and the government declared a general holiday. It was not possible to conduct face-to-face interviews for data collection during this period, as the population movement was restricted. Thus, the data were collected through the online survey portal, Google Forms, using Bengali as a language. A link to the form was then created and sent to the prospective participants, by e-mail, WhatsApp, or Facebook. All the participants to whom the survey link was sent were requested to share the link in their network to reach more people. The research team members circulated the survey link in their respective professional and social networks through the snowball process. As recommended by the WHO (19), the online data collection portal was active for 7 days. The respondents took an average of 20 min to complete the questionnaire. Though the initial decision was to reach a sample size of 1,000, a total of 1,059 respondents submitted their responses during these 7 days. However, three respondents did not consent to participate in this survey, and the final sample size was 1,056.

### Outcome Variables

#### Preparedness Toward Coronavirus Disease 2019

Preparedness is the state of readiness to prevent the spread of the COVID-19 (20). We assessed the preparedness toward COVID-19 by using eight Likert items (Table 1), and the response options for these items were “strongly disagree = 5,” “disagree = 4,” “neither agree nor disagree = 3,” “agree = 2,” and “strongly agree = 1.” We conducted a principal component analysis (PCA) by using these eight items. The PCA had an acceptable level of Kaiser–Meyer–Olkin (KMO) measure of sampling adequacy (KMO = 0.637). The varimax rotation with an eigenvalue higher than 1 was used as a selection criterion of components. The PCA produced four components that had an eigenvalue higher than 1. The eigenvalue of the first, second, third, and fourth components was 2.31, 1.75, 1.21, and 1.08, which explained 28.9, 21.9, 15.1, and 13.6% of the variance in the total items, respectively. These four components altogether explained 79.5% of the total items. The result of PCA indicates that the first two items, “washing my hands often with water and soap for 20 s each time is inconvenient for me” (19) and “disinfecting mobile phones, shoes, clothes each time I return home is inconvenient for me” (21, 22) (α = 0.809), were included under component 2, which was named “individual preparedness” (23). The third and fourth items, “keeping distance with family members will be difficult if they/I show COVID-19 related symptoms” and “keeping the older people in the house is challenging” (24) (α = 0.617), were included under component 3, which was named “household preparedness” (23). The fifth and sixth items, “due to the economic condition, I had to go to work though I am aware of the risk of COVID-19” and “I had to go out to save my job” (α = 0.868), were included under component 1, which was named “economic preparedness” (23, 25). The last two items, “most of the people of my locality do not maintain lockdown, so I also go out” and “go out for refreshment as I feel bored for staying at home for a few days” (α = 0.618), were included under component 4, which was named “community and social distancing-related preparedness” (26). We summed up the items of each component to create a continuous score for the preparedness scale about preventive practices against COVID-19, which ranges from 1 to 10, where the higher value indicated a higher level of preparedness.

**Table 1:** Rotated component matrix of principal component analysis of preparedness items against COVID-19 in Bangladesh.

**Items**	**Components**				**Responses**, ***n*** **(%)**		
	**1**	**2**	**3**	**4**	**Disagree^a^**	**Neither agree nor disagree**	**Agree^b^**
**Individual preparedness (α = 0.809)**							
Washing my hands often with water and soap for 20 s each time is inconvenient for me.		0.900			133 (12.6)	33 (3.1)	890 (84.3)
Disinfecting mobile phones, shoes, and clothes each time I return home is inconvenient for me.		0.919			257 (24.3)	54 (5.1)	745 (70.5)
**Household preparedness (α = 0.617)**							
Keeping distance with family members will be difficult if they/I show COVID-19-related symptoms.			0.837		264 (25.0)	56 (5.3)	736 (69.7)
Keeping the older people in the house is challenging.			0.838		328 (31.1)	77 (7.3)	651 (61.6)
**Economic preparedness (α = 0.868)**							
Due to the economic condition, I had to go to work, though I am aware of the risk of COVID-19.	0.923				431 (40.8)	69 (6.5)	556 (52.7)
I had to go out to save my job.	0.930				540 (51.1)	130 (12.3)	386 (36.6)
**Community and social distancing-related preparedness (α = 0.618)**							
Most of the people of my locality do not follow lockdown rules, so I also go out.				0.831	513 (48.6)	160 (15.2)	383 (36.3)
Go out for refreshment, as I feel bored for staying at home for a few days.				0.857	901 (85.3)	59 (5.6)	96 (9.1)

*COVID-19, coronavirus disease 2019*.

a “Disagree” includes both “disagree” and “strongly disagree.”

b “Agree” includes both “agree” and “strongly agree.”

### Independent Variables

There were limited independent variables in the study instrument, as the survey was conducted online. We included the following independent variables: age, sex, educational attainment, occupation, region, place of residence, marital status, knowing someone as COVID-19 positive among the respondent's immediate social environment, and respondent's COVID-19 status. We also used the knowledge (Supplementary Table 1), attitudes (Supplementary Table 2), and worriedness (Supplementary Table 3) scales related to COVID-19 as covariates. We assessed the knowledge related to COVID-19, using a total of 25 items. The response options of these items were “yes,” “no,” or “not sure/do not know.” We assigned 1 point to a correct response, while an incorrect response was assigned 0 points. The total score of these 25 items ranged between 0 and 25, with a higher score indicating better knowledge about COVID-19. The reliability analysis was performed to check the internal consistency of these 25 items and found an acceptable level of Cronbach alpha (α = 0.689). Attitudes are the way of feeling or thinking, while worriedness is the state of being worried or tensed. Attitudes toward COVID-19 (α = 0.671) and worriedness during COVID-19 (α = 0.813) were assessed using 8 and 10 Likert-type items, respectively. The response options for attitudes items were “strongly disagree = 1,” “disagree = 2,” “neither agree nor disagree = 3,” “agree = 4,” and “strongly agree = 5.” The scores of attitudes toward COVID-19 ranged between 8 and 40, where a higher score of these scales indicates higher negative attitudes. On the other hand, the response options for worriedness items were “do not worry at all = 1,” “worry sometimes = 2,” “worry often = 3,” and “worry all the time = 4.” The scores of the worriedness scale also ranged between 8 and 40, and a higher score of these scales indicates a higher worriedness during the period of COVID-19.

### Statistical Analysis

We first utilized univariate descriptive statistics [percentage, mean, and standard deviation (SD)] along with the accuracy test of each scale, where we divided the mean score of each scale by the total score of the respective scale. The independent sample *t*-test (if the independent variables had two categories), one-way ANOVA (if the independent variables had more than two categories), Pearson's product-moment correlation (if the independent variables were interval level), and Spearman's rank-order correlation (if the independent variables were ordinal) were used to produce the bivariate level statistics. We entered the statistically significant (*p* ≤ 0.05) variables at the bivariate level into the multiple linear regression model after checking the assumptions and multicollinearity. We used the Statistical Product and Service Solutions (SPSS) software, version 26, to analyze the data.

### Ethical Approval

The Bangladesh Medical Research Council approved the study (Registration Number: 302 1 1 05 2020). Participation in this online-based survey was entirely voluntary, and no incentives were provided to the participants. The respondents were informed about the aims, objectives, potential scopes, and implications of the findings of this study and were requested to participate voluntarily. As the data were collected through an online survey, the participants could only start filling up the questionnaire once they provided their consent to participate voluntarily.

## Results

### Background Characteristics

The average age of the respondents was 32 years, with an SD of 10.56 (Table 2). Most of the respondents were from the age group of 18–30 years (58.3%). About two thirds (65.2%) of the respondents were men, while the majority of the respondents (50.4%) had an education level up to a Master's degree. One in five respondents (20.5%) were professionals, while 38.5% of the respondents were students and unemployed. Nearly two thirds (63.9%) of the respondents lived in the Dhaka division, while 73.4% of the respondents were from the middle region of Bangladesh, and 66.9% of the respondents were from the city corporation area. The proportion of unmarried respondents was slightly higher (52.2%) than the married respondents. One third (32.8%) of the respondents knew someone as COVID-19 positive in their immediate social environment. However, none of the respondents were COVID-19 positive, though 2.2% felt that they might be carrying the coronavirus infection but did not get tested. Moreover, the average score for knowledge, attitudes, and worriedness related to COVID-19 was 17.1, 13.7, and 25.5, respectively.

**Table 2:** Background characteristics (%) and mean distribution of different types of preparedness against COVID-19 in Bangladesh.

**Background**	***n* (%)**	**Economic**	**Individual**	**Household**	**Community and social distancing**
		**Mean (SE)**	**Mean (SE)**	**Mean (SE)**	**Mean (SE)**
**Age**		***p*** **= 0.410**	***p*** **= 0.206**	***p*** **= 0.627**	***p*** **= 0.292**
18–24	341 (32.3)	6.70 (0.13)	7.61 (0.12)	5.07 (0.11)	8.20 (0.10)
25–30	275 (26.0)	6.42 (0.16)	7.57 (0.13)	4.87 (0.13)	8.47 (0.11)
31–39	184 (17.4)	6.31 (0.20)	7.73 (0.16)	5.11 (0.15)	8.33 (0.13)
40–49	178 (16.9)	6.33 (0.21)	7.93 (0.16)	5.07 (0.17)	8.42 (0.13)
50+	78 (7.4)	6.58 (0.29)	8.08 (0.22)	5.23 (0.28)	8.14 (0.17)
Mean (SD)	31.56 (10.56)	r = −0.02, *p* = 0.470	r = 0.06, *p* = 0.055	r = 0.02, *p* = 0.444	r = 0.01, *p* = 0.865
**Sex**		*p* ≤ 0.001	*p* = 0.123	*p* = 0.720	*p* = 0.001
Male	688 (65.2)	6.28 (0.10)	7.64 (0.09)	5.06 (0.08)	8.19 (0.07)
Female	368 (34.8)	6.87 (0.13)	7.85 (0.10)	5.01 (5.01)	8.57 (0.08)
**Education**		*p* = 0.050	*p* = 0.001	*p* = 0.013	*p* = 0.522
Up to higher secondary	82 (7.8)	6.76 (0.27)	7.24 (0.29)	5.78 (0.26)	8.26 (0.17)
Graduate	352 (33.3)	6.50 (0.13)	7.66 (0.11)	5.00 (0.11)	8.22 (0.10)
Masters	532 (50.4)	6.33 (0.11)	7.68 (0.09)	4.97 (0.09)	8.38 (0.08)
MPhil/PhD	90 (8.5)	7.09 (0.28)	8.53 (0.19)	4.91 (0.24)	8.44 (0.19)
**Occupation**		*p* ≤ 0.001	*p* = 0.004	*p* = 0.154	*p* = 0.278
Government and private sector job	181 (17.1)	5.71 (0.21)	7.39 (0.17)	5.25 (0.16)	8.44 (0.12)
Professional	211 (20.5)	6.82 (0.18)	7.95 (0.14)	4.93 (0.15)	8.41 (0.12)
NGO worker	173 (16.4)	6.53 (0.20)	8.13 (0.15)	4.90 (0.15)	8.46 (0.13)
Students and unemployed	407 (38.5)	6.7 (0.12)	7.57 (0.11)	4.97 (0.10)	8.20 (0.09)
Others	84 (8.0)	6.19 (0.26)	7.64 (0.25)	5.46 (0.24)	8.14 (0.18)
**Region**		*p* = 0.130	*p* = 0.098	*p* = 0.649	*p* = 0.001
Eastern (Sylhet and Chattaogram)	126 (11.9)	6.1 (0.22)	7.60 (0.20)	5.10 (0.18)	7.84 (0.16)
Middle (Dhaka, Barisal, and Mymensingh)	775 (73.4)	6.57 (0.09)	7.79 (0.08)	5.00 (0.08)	8.43 (0.06)
Western (Khulna, Rajshahi, and Rangpur)	155 (14.7)	6.37 (0.21)	7.40 (0.17)	5.17 (0.17)	8.15 (0.14)
**Place of residence**		*p* = 0.120	*p* ≤ 0.001	*p* = 0.031	*p* ≤ 0.001
Rural area	180 (17.0)	6.23 (0.18)	6.82 (0.19)	5.42 (0.18)	7.74 (0.15)
Urban (other than city corporation)	170 (16.1)	6.28 (0.20)	7.72 (0.16)	4.95 (0.16)	7.99 (0.14)
City corporation	706 (66.9)	6.6 (0.10)	7.94 (0.07)	4.96 (0.08)	8.55 (0.06)
**Marital status**		*p* = 0.030	*p* = 0.476	*p* = 0.608	*p* = 0.487
Married	505 (47.8)	6.31 (0.12)	7.76 (0.10)	5.07 (0.10)	8.36 (0.08)
Unmarried	551 (52.2)	6.65 (0.10)	7.67 (0.09)	5.01 (0.09)	8.29 (0.08)
**Know someone as infected with the COVID-19 in their immediate social environment**		*p* = 0.230	*p* = 0.028	*p* = 0.560	*p* = 0.009
No	710 (67.2)	6.42 (0.10)	7.61 (0.08)	5.06 (0.08)	8.22 (0.07)
Yes	346 (62.8)	6.62 (0.14)	7.92 (0.11)	4.98 (0.11)	8.53 (0.09)
**Knowledge related to symptoms and transmission of COVID-19**	16.92 (3.29)	r = 0.03, *p* = 0.300	r = 0.06, *p* = 0.038	r = −0.02, *p* = 0.622	r = 0.05, *p* = 0.146
**Attitudes toward COVID-19**	13.72 (3.69)	r = −0.11, *p* ≤ 0.001	r = −0.14, *p* ≤ 0.001	r = 0.05, *p* = 0.096	r = −0.18, *p* ≤ 0.001
**Worriedness about COVID-19**	25.46 (5.420	r = −0.10, *p* ≤ 0.001	r = 0.03, *p* = 0.375	r = −0.16, *p* ≤ 0.001	r = −0.05, *p* = 0.147
**Total**	1,056 (100.0)	6.49 (0.08)	7.71 (0.07)	5.04 (0.07)	8.32 (0.05)

*COVID-19, coronavirus disease 2019; NGO, non-governmental organization*.

### Prevalence of Preparedness Related to Coronavirus Disease 2019

Table 1 presents the distribution of the statements used to assess the preparedness of the respondents about preventive practices against the COVID-19. The mean score of the total preparedness scale was 27.6, with an SD of 4.7, and an overall preparedness level was 68.9% (27.55/40 ^*^ 100). Nearly a quarter of the respondents agreed that disinfecting daily-use commodities such as mobile phones, shoes, and clothes each time they return home was inconvenient for them, while 61.6% respondents agreed that it was challenging for them to keep the older people in the house as part of the prevention of COVID-19. Nearly half of the respondents (52.7%) agreed that they had to go to work due to their economic condition, and 36.6% of the respondents reported that they went out of their home, as most of the people of their locality did not follow the lockdown rules.

### Differentials and Associates of Preparedness Related to Coronavirus Disease 2019

Table 2 shows that the mean score related to economic preparedness, individual preparedness, household preparedness, and community and social distancing-related preparedness was 6.49, 7.71, 5.04, and 8.32, respectively. The economic preparedness score was statistically significantly varied by sex, education, occupation, marital status, attitudes, and worriedness of COVID-19. The individual preparedness score was statistically significantly varied by education, occupation, place of residence, and knowing someone infected with COVID-19 in the respondent's immediate social environment. The household preparedness score was statistically significantly varied by education, place of residence, and worriedness of COVID-19. On the other hand, community and social distancing-related preparedness scores were statistically significantly varied by sex, region, place of residence, and knowing someone infected with the COVID-19 in the respondent's immediate social environment. Besides, higher knowledge related to symptoms and transmission of COVID-19 was statistically significantly correlated with higher individual preparedness, while higher negative attitudes toward COVID-19 was significantly negatively correlated with economic, individual, and community and social distancing-related preparedness. Finally, a higher level of worry about COVID-19 was statistically significantly correlated with lower levels of economic and household-related preparedness.

We entered the significant variables at the bivariate levels into the multiple linear regression models after checking the assumptions and multicollinearity. The age of the respondent was highly correlated with education (r = 0.693, *p* ≤ 0.001) and marital status (r = 0.761, *p* ≤ 0.001); thus, age was excluded from multiple regression analyses. Table 3 presents the standardized beta coefficients of multiple linear regression with their statistical significance. It shows that women had mean 0.091-unit higher economic preparedness than men (β = 0.091, *p* = 0.003); other variables held constant. The respondents who worked in the government and private sectors had significant mean 0.113-unit lower economic preparedness (β = −0.113, *p* = 0.008) than the students and unemployed. The 1-unit increase of negative attitudes toward COVID-19 (β = −0.083, *p* = 0.008) and worriedness during COVID-19 (β = −0.103, *p* = 0.001) were decreasing the mean economic preparedness by 0.083 and 0.103 units, respectively. These predictors explained a 5.5% variation of the total model.

**Table 3:** Association between background characteristics and different types of preparedness against COVID-19 in Bangladesh.

**Background characteristics**	**Economic**	**Individual**	**Household**	**Community and social distancing**
**Sex** (Ref: Male)				
Female	0.091^**^			0.077^*^
**Educational attainment** (Ref: Up to higher secondary)				
Undergraduate	−0.065	0.072	−0.157^**^	
Post-graduate (Masters)	−0.036	0.044	−0.171^**^	
MPhil/PhD	0.038	0.120^**^	−0.101^*^	
**Occupation** (Ref: Students and unemployed)				
Government and private sector	−0.113^**^	−0.063		
Professional	0.012	−0.011		
NGO worker	−0.023	0.038		
Others	−0.053	0.002		
**Region** (Ref: Eastern)				
The middle part of Bangladesh				0.070
The western part of Bangladesh				0.088^*^
**Place of residence** (Ref: Rural)				
Urban (other than city corporation)		0.128^**^	−0.081^*^	0.023
City corporation		0.220^**^	−0.083^*^	0.161^**^
**Marital status** (Ref: Married)				
Unmarried	0.053			
**Know someone as COVID-19 positive within their immediate social environment**				
Yes (Ref: No)		0.028		0.047
**Knowledge related to COVID-19**		0.016		
**Attitudes toward COVID-19**	−0.083^**^	−0.081^**^		−0.144^**^
**Worriedness related to COVID-19**	−0.103^**^		−0.167^**^	
**Constant^a^**	7.830^**^	7.232^**^	7.341^**^	8.057^**^
**Model summary**				
*N*	1,056	1,056	1,056	1,056
R	0.235	0.254	0.208	0.262
R^2^	0.055	0.065	0.043	0.069
Adjusted R^2^	0.045	0.052	0.037	0.062

*COVID-19, coronavirus disease 2019; NGO, non-governmental organization*.

* *p ≤ 0.05*.

** *p ≤ .01*.

a *Unstandardized beta*.

Table 3 also shows that the mean individual preparedness was 0.120 units higher among the respondents who had an MPhil/PhD level of education (β = 0.120, *p* = 0.009) than the respondents who had up to higher secondary level of education. Similarly, other things held constant, the urban respondents (β = 0.128, *p* = 0.001) and the city corporation area respondents (β = 0.220, *p* ≤ 0.001) had 0.128- and 0.220-unit higher mean individual preparedness than the rural respondents. Besides, the negative attitudes toward COVID-19 had a negative influence (β = −0.081, *p* = 0.010) on individual preparedness. The independent variables of this model explained 6.5% of the variation of this model.

The mean household preparedness was 0.157, 0.171, and 0.101 units lower among the respondents with undergraduate (β = −0.157, *p* = 0.006), postgraduate (β = −0.171, *p* = 0.004), and MPhil/PhD (β = −0.101, *p* = 0.018) levels of education than that of higher secondary level. The respondents living in the urban areas (β = −0.081, *p* = 0.039) and the city corporation areas (β = −0.083, *p* = 0.041) had lower mean household preparedness than the respondents living in rural areas. It was also observed that the 1-unit increase of the worriedness related to COVID-19 decreased the mean household preparedness by 0.167 units (β = −0.167, *p* ≤ 0.001). These predictors explained a 4.5% variation of the total model.

The mean community and social distancing-related preparedness was 0.077 units higher among women than men (β = 0.077, *p* = 0.013). The respondents living in the western part of Bangladesh had 0.088 units higher mean community and social distancing-related preparedness (β = 0.088, *p* = 0.033) than those in the eastern part. Similarly, compared with the respondents living in rural areas, the respondents living in the city corporation areas (β = 0.161, *p* ≤ 0.000) had 0.161 units higher preparedness. In contrast, 1-unit increment of the attitudes toward COVID-19 had negatively influenced (β = −0.144, *p* ≤ 0.001) the community and social distancing-related preparedness by 0.144 units. These regressors explained around 6.9% of the total variation of the model.

## Discussion

The study sought to explore the preparedness regarding preventive practices against COVID-19 in Bangladesh. The study found that the overall preparedness level was 68.9% (27.56/40 ^*^ 100).

### Individual Preparedness

The level of individual preparedness for preventing practices against COVID-19 was 77.1% (7.71/10 ^*^ 100). The findings show that 12–24% of respondents reported their inconvenience regarding proper handwashing practices and disinfecting items of personal use after each time they return home. This inconvenience related to personal hygiene could be attributed to factors like the availability of handwashing commodities, price, facilities, and knowledge and attitudes toward handwashing (27–30).

This study found that individual preparedness was higher among the respondents who had MPhil/PhD level of education, which is similar to the studies conducted elsewhere (23, 31). The relation between education and individual preparedness creates health communication scope among the mass population with the utmost importance (32, 33). Findings regarding other recent infectious disease outbreaks in Bangladesh (dengue, chikungunya, Nipah virus, etc.) also indicate that mass population's knowledge level and preventive practices amidst disease outbreaks are significantly associated (34). The findings of this study showed that the respondents living in the urban and the city corporation areas had higher individual preparedness than the respondents living in rural areas. The urban populations are in an advantageous position because they are more likely to afford and have access to personal hygiene-related amenities (32).

The negative attitudes toward COVID-19 were producing less individual preparedness. This finding is consistent with the Chinese study (6). Our study measured negative attitudes toward COVID-19 by using items like COVID-19 is a punishment from the creator and we (respondents) can be safe if we pray to Allah/God/Creator regularly. These attitudes possibly reduced the risk perception (35) of the respondents, which push them to be less prepared (36).

### Household Preparedness

The level of household preparedness for preventing practices against COVID-19 was 50.4% (5.04/10 ^*^ 100). Around two thirds of the respondents reported their inconvenience in keeping older persons in the house and maintaining social distance with family members showing symptoms related to COVID-19. Maintaining social distancing with family members, especially with older persons within the home setting, was also challenging in other studies (37–40).

The household preparedness was found lower among the respondents with undergraduate, postgraduate, and MPhil/PhD levels of education than higher secondary levels. This finding needs to be interpreted with the fact that more respondents with higher secondary level education were living in rural areas while higher educated respondents were living in urban and city corporation areas. The housing pattern of the rural and urban areas is structurally different (41), and urban housing in Bangladesh lacks the comfortability for the older people. This finding is supported by the findings that the respondents living in urban areas had lower household preparedness. It was challenging for many city dwellers to maintain social distancing at home in the densely populated cities and the congested housing system (2, 42, 43). It was also observed in the current study that the higher the worriedness related to COVID-19, the lower the household preparedness would be. The adverse impacts of COVID-19 imposed social isolation, be it physical or psychological, may lead people to be less willing to isolate family members (44, 45), including older persons, even if they show related symptoms.

### Economic Preparedness

The level of economic preparedness for preventing practices against COVID-19 was 64.9% (6.49/10 ^*^ 100). More than half of the respondents reported economic consideration as their motive to go outside of the home, whereas more than one third of the respondents reported saving jobs as their prioritized concern even in the lockdown period. Financial fears have also been reported in other studies as the main motive for going outside in the present context (46). Working-class people were less likely to comply with the lockdown protocols because of their economic urgency and drive to save jobs (47, 48).

The findings of this study showed a more secure economic position and higher preparedness among women and students, and the unemployed. The economic reliance of these subgroups on men and employed family members contributed to their better-secured position and better economic preparedness. The financial fear and perceived insecure position among employed respondents were reported in other studies, too (23, 49). The effect of negative attitudes toward COVID-19 and worriedness negatively influenced economic preparedness in this study.

### Community and Social Distancing-Related Preparedness

The level of preparedness related to community and social distancing was 83.2% (8.32/10 ^*^ 100). In this regard, respondents who were women and living in the city corporation areas were found to be more prepared. Women's higher perception and better compliance regarding community and social distancing-related preparedness were also found in other studies (50, 51). The rural respondents were also found to be showing poor social distancing patterns, which has been supported in another study as well (52). The negative attitudes toward COVID-19 were found to be having a negative influence on the social distancing-related preparedness, as negative attitudes possibly reduced the risk perception of the respondents, which push them to be less prepared (53, 54).

### Conclusions and Implications

In a context where a better preparation level and evidence-based preventive practices can make things more comfortable, this study found an overall preparedness level of 68.9%, which significantly implies scopes of priority-based interventions. Specific preparedness levels concerning economic (64.9), individual (77.1), household (50.4), community, and social distance (83.2) aspects also are supporting the necessity of the above-mentioned implications. Inconvenience regarding ensuring personal hygiene-related practices was reported, which reflected the lack of individual-level preparedness. Maintaining social distance was very challenging, which was significantly influenced by the presence of negative attitudes toward COVID-19. Protection of the older population who are “the most at-risk population” by successfully making them stay within the house faces challenges too because of their particular contexts. Financial urgency, including the drive to save jobs, was seen to triggering mass population's tendency to not follow the rules of lockdown and social distancing. The findings of this study implicate the necessity of taking comprehensive efforts to ensure the coverage and receipt of the different social protection schemes, especially for older persons, to release them from the financial fears and urgency to go outside amidst the coronavirus period. A fixed amount of financial compensation, especially toward economically vulnerable groups who are found to be not following lockdown rules for the drive to save job, can also be considered in this regard to provide them with temporary support and also enable them to sustain their daily lives under financial protection. Policy interventions to increase individual awareness have been observed to be effective in creating preventive behaviors and preventing infectious diseases in incidents of other outbreaks in the context of Bangladesh (55). Thus, the findings of this study can be applied to the broader context of infectious disease-related disaster preparedness, such as dengue, chikungunya, and Nipah virus. The current study also implicates the necessity of ensuring the broader coverage of health education related to personal hygiene practices to increase the level of awareness through appropriate channels, particularly in the rural areas where the level of individual preparedness was lower.

### Strength and Limitations

This study provided efforts to explore the preparedness regarding the preventive practices of the mass population against COVID-19 with a broad geographical coverage within a short period. Such rapid snapshots with robust statistical analyses can provide food for thought for the policy planners. However, a rapid assessment survey to understand the preparedness regarding the preventive practice of the mass population in Bangladesh regarding COVID-19 clearly has certain limitations. First, this was an online survey, and it covered somewhat a homogenous population in terms of knowledge and skills, and level of awareness regarding health issues. Thus, these findings have certain limitations in establishing generalizability. Second, as it was a rapid assessment online-based survey, the study team had to take the time issue (required minutes to fill up the questionnaire) of the respondents into consideration, and it left scopes for reaching depth with potential items to use to assess the preparedness level and preventive practice in a better way. Third, the sample size used in this study varied greatly across different divisions. The imbalanced sample size may cause bias in the study findings. Besides, some essential covariates were not included in the questionnaire due to the online nature of the survey. Finally, this study leaves ample room for further exploration of the population level preparedness and its relevance with the recurrent infectious disease outbreaks in the context of Bangladesh.

## Data Availability Statement

The raw data supporting the conclusions of this article will be made available by the authors, without undue reservation.

## Ethics Statement

The studies involving human participants were reviewed and approved by Bangladesh Medical Research Council. The patients/participants provided their written informed consent to participate in this study.

## Author Contributions

MBH, MA, MI, SS, and MF conceptualized the study. MBH, MA, MI, SS, MF, SR, MM, MAH, SK, AM, HM, and SSS designed the study and collected the data. MA, MI, and SS analyzed the data with guidance from MBH. MA, SS, and MBH interpreted the data and drafted the manuscript. MI, MF, SR, MM, MAH, SK, AM, HM, and SSS revised the manuscript critically for valuable intellectual content and approved the final version to be published. All authors remain in agreement to be accountable for all aspects of the work.

## Conflict of Interest

The authors declare that the research was conducted in the absence of any commercial or financial relationships that could be construed as a potential conflict of interest.
