# Supplementary material for: Population-Level Preparedness About Preventive Practices Against Coronavirus Disease 2019: A Cross-Sectional Study Among Adults in Bangladesh
Source: Front Public Health. 2021 Jan 11;8:582701. doi: 10.3389/fpubh.2020.582701 (PMC7832348; doi:10.3389/fpubh.2020.582701)
Supplement: Supplementary file 1 [file Data_Sheet_1.PDF]

Supplementary Tables

STable 1. Items used to measure knowledge related to COVID-19

Knowledge related to COVID-19	n (%)	
	**Correct knowledge**	**Incorrect knowledge**
**Knowledge about symptoms of COVID-19**		
**Most common symptoms**		
Fever	1022 (96.8)	34 (3.2)
Dry cough	1002 (94.9)	54 (5.1)
Fatigue (tiredness)	697 (66.0)	359 (34.0)
**Less common symptoms**		
Muscle or body aches and pains	701 (66.4)	355 (33.6)
Nasal congestion	718 (68.0)	338 (32.0)
Sore throat	1002 (94.9)	54 (5.1)
Diarrhoea	741 (70.2)	315 (29.8)
Conjunctivitis	144 (13.6)	912 (86.4)
Headaches	616 (58.3)	440 (41.7)
Loss of taste or smell	568 (53.8)	488 (46.2)
A rash on the skin, or discoloration of fingers or toes	269 (25.5)	787 (74.5)
**Serious symptoms**		
Shortness of breath	1026 (97.2)	30 (2.8)
Chest pain or pressure	574 (54.4)	482 (45.6)
Loss of speech or movement	60 (5.7)	996 (94.3)
The novel Coronavirus can be asymptomatic	973 (92.1)	83 (7.9)
**Knowledge about treatment and vaccine of Coronavirus**		
There is no drug to treat the novel Coronavirus	772 (73.1)	284 (26.9)
There is no vaccine for the novel Coronavirus	978 (92.6)	78 (7.4)
**Knowledge about transmission and incubation period of Coronavirus**		
The novel Coronavirus can be transmitted by animals to humans only	270 (25.6)	786 (74.4)
The novel Coronavirus is transmissible via droplets through coughing, sneezing or intimate contact	1031 (97.6)	25 (2.4)
The novel Coronavirus can remain alive for more than four hours	640 (60.6)	416 (39.4)
The novel Coronavirus can transmit during sexual intimacy	462 (43.8)	594 (56.3)
The novel Coronavirus can transmit through papers and cartoons used in packing groceries/foods/packets that we order online	817 (77.4)	239 (22.6)
A Coronavirus infected person can be recovered from COVID-19	1010 (95.6)	46 (4.4)
A recovered person be infected with Coronavirus again	859 (81.3)	197 (18.7)
What is the incubation period of the novel coronavirus?	918 (86.9)	138 (13.1)
**Average score for knowledge related to COVID-19 = 17.1 (SD = 3.10)**		

STable 2. Items used to measure attitudes toward COVID-19

Statements	n (%)		
	Strongly disagree and disagree	Neutral	Agree and strongly agree
Covid-19 is a human-made disease	443 (42.0)	446 (42.2)	167 (15.8)
Positive with the Novel Coronavirus means death is definite	1018 (96.4)	29 (2.7)	9 (0.9)
Covid-19 is a punishment from the creator	473 (44.8)	322 (30.5)	261 (24.7)
Covid-19 does not attack Muslim people	1035 (98.0)	18 (1.7)	3 (0.3)
Non-Muslims are more prone to be infected by this virus	948 (89.9)	61 (5.8)	47 (4.5)
There is nothing called Coronavirus; it is just a bad air	1024 (97.0)	26 (2.5)	6 (0.6)
We can be safe if we pray to Allah/God/Creator regularly	752 (71.2)	193 (18.3)	111 (10.5)
Coronavirus is created by Media	990 (93.8)	51 (4.8)	15 (1.4)
**Average score for attitude toward COVID-19 = 13.7 (SD = 3.7)**			

STable 3. Items used to measure worriedness toward COVID-19

**Statements**	**Do not worry at all**	**Worry sometimes**	**Worry often**	**Worry all the time**
Losing someone I love	147 (13.9)	387 (36.6)	334 (31.6)	188 (17.8)
Own health	185 (17.5)	559 (52.9)	236 (22.3)	76 (7.2)
Family member's health	35 (3.3)	351 (33.2)	417 (39.5)	253 (24)
Not being able to pray in the mosque	531 (50.3)	290 (27.5)	166 (15.7)	69 (6.5)
Health system getting overloaded	58 (5.5)	345 (32.7)	433 (41)	220 (20.8)
Shut down of educational institutions	226 (21.4)	339 (32.1)	340 (32.2)	151 (14.3)
Economic recession	21 (2)	264 (25)	438 (41.5)	333 (31.5)
Restricted access to food supplies	63 (6)	370 (35)	425 (40.2)	198 (18.8)
Society is getting more self-centered	212 (20.1)	368 (34.8)	317 (30)	159 (15.1)
Risk of famine	91 (8.6)	308 (29.2)	379 (35.9)	278 (26.3)
**Average score for worriedness related COVID-19 = 25.5 (SD = 5.4)**				
